# Neurological Consequences, Mental Health, Physical Care, and Appropriate Nutrition in Long-COVID-19

**DOI:** 10.1007/s10571-022-01281-w

**Published:** 2022-09-14

**Authors:** Pasquale Picone, Tiziana Sanfilippo, Rossella Guggino, Luca Scalisi, Roberto Monastero, Roberta Baschi, Valeria Mandalà, Livio San Biagio, Manfredi Rizzo, Daniela Giacomazza, Clelia Dispenza, Domenico Nuzzo

**Affiliations:** 1 Istituto per la Ricerca e l’Innovazione Biomedica, Consiglio Nazionale delle Ricerche, Via U. La Malfa 153, 90146 Palermo, Italy; 2 Dipartimento di Scienze e Tecnologie Biologiche, Chimiche e Farmaceutiche-STEBICEF, Università degli Studi di Palermo, 90128 Palermo, Italy; 3 Presidio Ospedaliero “S. Cimino”, Anestesia e Rianimazione, 90141 Termini Imerese, Palermo, Italy; 4 Ambulatorio di Nutrizione Clinica ASP Palermo, Via G. Cusmano 24, 90141 Palermo, Italy; 5 Centro Medico di Fisioterapia “Villa Sarina”, Via Porta Palermo, 123, 91011 Alcamo, Italy; 6 Department of Biomedicine, Neuroscience and Advanced Diagnostics, University of Palermo, Via La Loggia 1, 90129 Palermo, Italy; 7 Regional Register of Psychologists (OPRS), Via G.M. Pernice, 5, 90144 Palermo, Italy; 8 UOC Cardiochirurgia, Azienda Ospedaliera Universitaria Integrata di Verona, Piazzale Stefani, 1, 37126 Verona, Italy; 9 Department of Health Promotion, Mother and Child Care, Internal Medicine and Medical Specialties, University of Palermo, 90133 Palermo, Italy; 10 Istituto di Biofisica, Consiglio Nazionale delle Ricerche, Via U. La Malfa 153, 90146 Palermo, Italy; 11 Dipartimento di Ingegneria, Università Degli Studi di Palermo, Viale delle Scienze, Bldg 6, 90128 Palermo, Italy

**Keywords:** Long-COVID-19, Nutrition and physical activity in long-COVID-19, Long-COVID-19 neurological implications, Psychological care in long-COVID-19 patients

## Abstract

SARS-CoV-2 pandemic has caused a collapse of the world health systems. Now, vaccines and more effective therapies have reversed this crisis but the scenario is further aggravated by the appearance of a new pathology, occurring as SARS-CoV-2 infection consequence: the long-COVID-19. This term is commonly used to describe signs and symptoms that continue or develop after acute infection of COVID-19 up to several months. In this review, the consequences of the disease on mental health and the neurological implications due to the long-COVID are described. Furthermore, the appropriate nutritional approach and some recommendations to relieve the symptoms of the pathology are presented. Data collected indicated that in the next future the disease will affect an increasing number of individuals and that interdisciplinary action is needed to counteract it.

## Introduction

During the SARS-CoV-2 pandemic, healthcare systems were worldwide under massive stress and emergency. SARS-CoV-2 has had and still continues to have an enormous impact on the population with negative consequences on various life areas. The disease presents a wide spectrum of clinical phenotypes ranging from asymptomatic infection to respiratory failure and multiorgan dysfunction. A very recent hypothesis explaining the varied clinical landscape found in COVID-19-affected people refers to innate immunity, the first ancestor defense against pathogens and virus, and in particular to the innate immunity humoral harm (Stravalaci et al. 2022). The latter consists of soluble Pattern Recognition Molecules (PRMs) having an antibody-like action and including the Mannose Binding Lectin (MLB) protein. Researchers determined that MLB was able to bind to the Spike protein of the different SARS-CoV-2 variants and inhibited the different virus species in vitro. The authors concluded that genetic polymorphisms at the level of MLB gene were associated with different COVID-19 severities and that MLB can be used as disease marker to predict the seriousness of the pathology (Stravalaci et al. 2022).

**Fig. 1:**
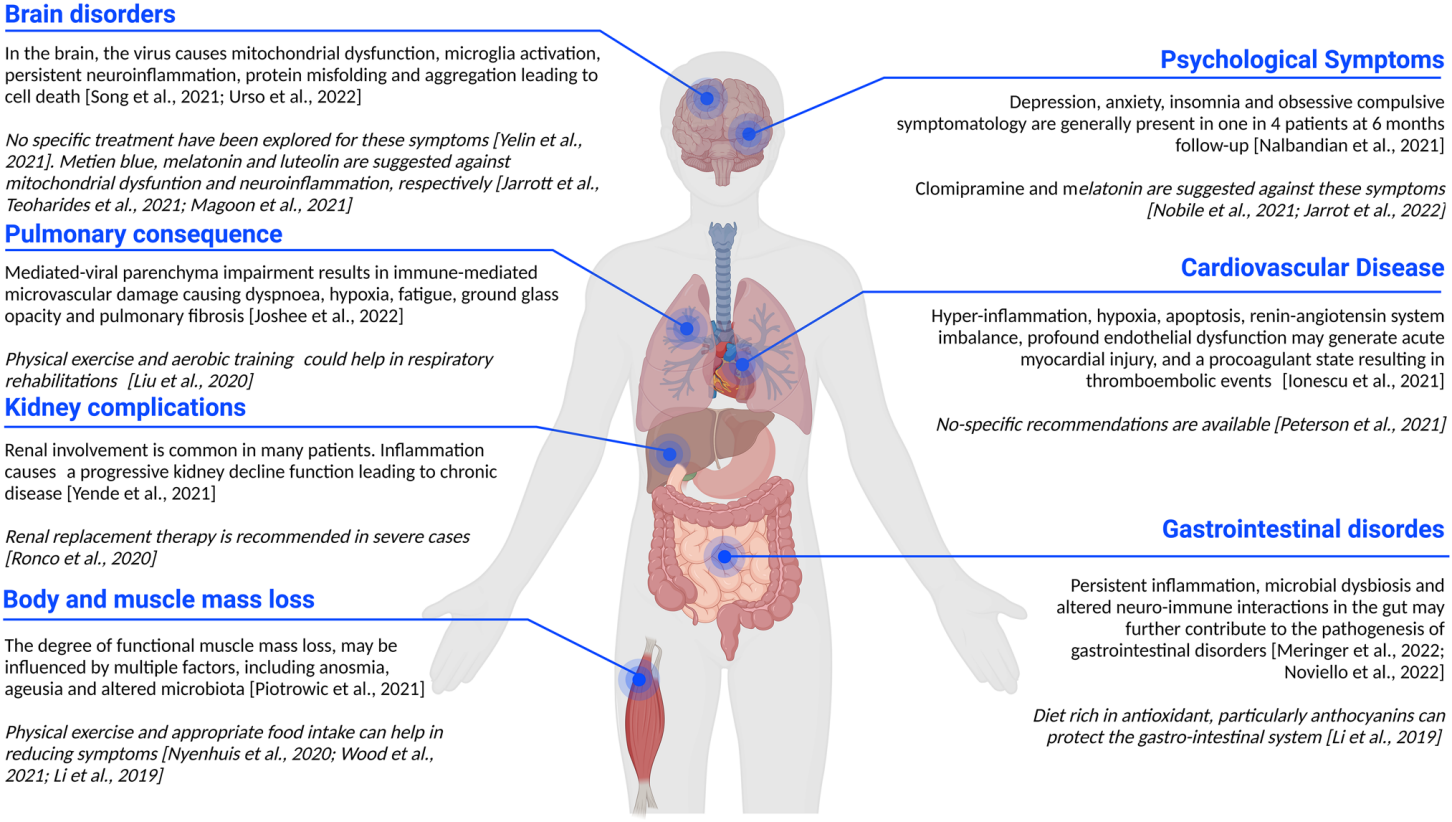
Long-COVID is a pathology interesting multiple organs. Gastrointestinal and neurological disorders, loss of body weight, and muscle mass can be observed. The symptoms also extend to the psychological sphere. Kidneys and cardiocirculatory disorders due to long-COVID-19 are not treated in the present review. Image created with BioRender.com

Scientists are increasingly concerned by the social and psychological implications that the diffusion of the virus has brought with it. Furthermore, it is now scientifically established that residual consequences of SARS-CoV-2 infection present after negative swab can occur, not rare enough to induce physicians and scientists to identify a new pathology called long-COVID-19 (hereafter reported as “long-COVID”). Symptoms, which can affect multiple organs are fatigue, loss of weight and muscle strength, dyspnea, chest pain, cognitive disturbances, arthralgia, myocardial inflammation, vessel and kidney damages, which can lead to reduced quality of life (Ionescu et al. 2021; Joshee et al. 2022; Peterson et al. 2021; Ronco et al. 2020; Urso et al. 2022; Yende and Parikh 2021; Nuzzo and Picone 2020; Nuzzo et al. 2021a); in the most severe cases, encephalitis can develop with inherent prognostic issues (Nuzzo et al. 2021b) (Fig. 1). Long-COVID affects a significant fraction (more than 50%) of COVID-19 patients, regardless of the severity of the pathology and recent research indicated that it can be more frequent in non-hospitalized patients (Mohamed-Hussein et al. 2021). Furthermore, if the respiratory symptoms are predominant in the acute phase of the disease, shortness of breath and neurological disorders are the main distinctive tract of the COVID-19 sequelae (Mohamed-Hussein et al. 2021). Today clinical evidence is evolving on the subacute and long-term effects of COVID-19 (Nuzzo et al. 2021b). The duration and severity of the symptoms after infection largely remain unknown. Many terms have been adopted to describe the prolonged manifestation following SARS-CoV-2 infection and its symptoms. Names such as “post-acute COVID-19,” “persistent COVID-19 symptoms,” “post-COVID-19 manifestations,” “long-COVID-19,” “long-term COVID-19 effects,” “post-COVID-19 syndrome,” “ongoing COVID-19,” “chronic COVID-19,” and “long-term sequelae” have been proposed in the past. Recently, “post-acute sequelae of SARS-CoV-2 infection” (PASC) and “long-COVID-19” have been generally accepted to define the disease.

Based on the recent discoveries, it is possible to distinguish the long-COVID-19 pathological scenario into two different classes. The first one concerns the post-COVID-19 condition or subacute symptomatic, which includes symptoms disappearing in 4 to 12 weeks after the acute COVID-19; the second one is the long-COVID-19 syndrome, which includes persistent symptoms still present up to 6 months or more—some studies report that only about 23% of the patients are completely symptom-free after 12 months (Seeßle et al. 2021) from the onset of the acute COVID-19 pathology and not diagnosable as other pathologies. Long-lasting symptoms may represent a continuum with those of the acute phase, thus long-COVID-19 has a beginning but not a definite end (Seeßle et al. 2021). However, monitoring the pathology is essential to define time boundaries and, in particular, the end of symptoms. Individual symptoms can last for varying time intervals, some may regress, others persist. Long duration of recovery appears to be related to high severity, but has also been reported regardless of the acute phase severity, hospitalization, and ICU admission.

It is known that SARS-CoV-2 infection is accompanied by a violent inflammatory response causing the release of a large amount of pro-inflammatory cytokines, the so-called “cytokine storm.” This event is characterized by excessive activation of inflammatory cells such as T cells, mast cells, and macrophages with the production and release of cytokines and chemical mediators (Mangalmurti and Hunter 2020). Mast cells are particularly interesting due to their action in the regulation of the other inflammatory cells. In normal cases, the sustained levels of pro-inflammatory cytokines is an appropriate response to protect the organs from infections; on the contrary, aberrant response causes organ failure and severe disease (Braciale and Hahn 2013), as in the case of SARS-CoV-2 infection. A recent hypothesis explaining the long-COVID-19 insurgence supposes that the mast cell activation syndrome could be at the basis of the disease (Afrin et al. 2020). This could also elucidate the different, from asymptomatic to severe, manifestations of the COVID-19 disease (Afrin et al. 2020). It should also be noted that mast cells express ACE2 receptors and for this reason, they can host SARS-CoV-2.

Taken together, COVID-19 and long-COVID patients represent a huge segment of the population. Systematic studies to develop a multidisciplinary approach to take care of these patients at home, for their greater comfort, are crucial and this could also represent a necessary way to avoid hospital overcrowding. To this purpose, a comprehensive understanding of patient needs after the acute phase of the illness will help in the development of in-home structure for long-COVID treatment. To this aim, a multidisciplinary strategy is to be adopted in the home management of patients with long-COVID in order to be able to shorten the different functional, cognitive, psychological, and behavioral consequences of the disorder. Furthermore, this approach must be tailored to the individual characteristics of a patient (e.g., comorbidity, polypharmacy, nutritional status, pre-morbid personality traits, pre-existent cognitive impairment and physical disability, etc.). For these reasons, an appropriate diet, cognitive and psychological support/rehabilitation, and specific exercise training and rehabilitation programs may be useful for a rapid improvement of the long-COVID syndrome.

## Neurological Consequences of Long-COVID-19

The evaluation of neurological manifestations in post-acute COVID-19 disease can be explained by the different populations taken as samples to address the problem. The COVID-19 is a very new disease and, at present, incomplete knowledge of the mechanism of infection and the complexity of viral and non-viral factors involved in the disease explain the incomplete information on the effects of the SASR-CoV-2 virus on the nervous system (Beghi et al. 2022). Although the main symptoms of COVID affect the respiratory system, acute and subacute neurological manifestations during the infection have been described in over 35% of cases (Nolen et al. 2022). Most frequently reported neurological diagnoses during the acute COVID-19 infection include ischemic and hemorrhagic stroke, delirium/encephalopathy, seizures, neuropathy, and myopathy. Persistent neurologic symptoms have been described in long-COVID subjects, who often described cognitive impairment/brain fog, headache, sensory symptoms, myalgias (Nolen et al. 2022), and verbal fluency deficit (Braga et al. 2022).

Long-COVID can be seen also in non-hospitalized subjects, the so-called *long haulers*, who experienced persistent and mild neurological symptoms. Lastly, rare and insidious para-infectious complications of COVID-19 infection may include acute demyelinating encephalomyelitis, acute necrotizing encephalopathy, and acute inflammatory demyelinating polyneuropathy (Nolen et al. 2022). The evolution of the neurological symptoms has been very recently investigated in a group of 52 patients, 27 SARS-CoV-2 positive non-hospitalized *long haulers* and 25 SARS-CoV-2 negative, from May to November 2020 (Ali et al. 2022). Patients did not show significant change in the neurological symptoms between the first and the last medical evaluation. Most of them still experienced brain fog, headache, dizziness, blurred vision, tinnitus, and fatigue. If on one hand, dysgeusia and anosmia had statistically improved, gastrointestinal and cardiocirculatory symptoms, memory impairment and attention had worsened in the last evaluation. In general, the quality of life was lower than that of the healthy population (Ali et al. 2022; Shanley et al. 2022). Vaccination did not have any impact on cognitive function and fatigue symptoms. Another recent study was conducted on 50 SARS-CoV-2 confirmed patients, 21 hospitalized and 29 non-hospitalized (Bungerberg et al. 2022). The average time from infection detection to investigation was about 30 weeks. Mild deficits were found in both groups in executive functions, fatigue, reduced performance in attention, and memory. Reduced sleep quality, increased anxiety, and depression were prevalent in non-hospitalized patients. Microbleeds were detected exclusively in non-hospitalized patients (Bungerberg et al. 2022). Neurocognitive disorder (MCD) in post-acute COVID-19 is a complex mechanism involving pre-existing comorbidities, such as the conditions during hospitalization, hypoxia due to pulmonary injury, and vascular and neuronal damages. Depression, anxiety, and fatigue, symptoms very often associated with long-COVID-19, were not found connected to MCD (Andriuta et al. 2022; Alonso-Lana et al. 2020; Almeria et al. 2020). A very comprehensive research has been recently published by Taquet and Coworkers (Taquet et al. 2022). They performed a 2-year study on about 1.3 million patients. Among them 14% were children, 18% older adults, 58% female; the average age was 42.5 years. Data were also compared with an equal number of patients with other respiratory disease. Researchers found that, while common psychiatric disorders returned to the baseline after 1–2 months, brain fog, dementia, psychotic disorders, epilepsy, and seizures were augmented at the end of the 2-year observation time. The alpha variant infection did not induce any significant different risk profiles between adults and children groups. The scenario changed with the delta and omicron variant infections; in these cases, cognitive problems, insomnia, and intracranial hemorrhage were prevalent in children. The result of the study is very useful to predict sequelae problems correlated to SARS-CoV-2 infection (Taquet et al. 2022). Furlan Damiano and Collaborators found a positive correlation between chemosensory problems (hypogeusia and parosmia) and cognitive dysfunction performing a neurobiological study on a statistical sample composed by 700 hospitalized mild-to-severe COVID-19 patients. The latter authors hypothesized that upon reaching the CNS, SARS-CoV-2 induces molecular and cellular cascade processes similar to those occurring in neurodegenerative diseases (Furlan Damiano et al. 2022).

According to the wide spectrum of the central nervous system (CNS) involvement during COVID-19 infection, the European Academy of Neurology organized a task force to manage the COVID-19 sequelae targeting CNS, suggesting specific guidelines for treatment of neurological symptoms and overall management of neurological complications during the infection. From a pathophysiological point of view, two main routes through which SARS-CoV-2 reaches the CNS have been hypothesized: direct and indirect (Nuzzo and Picone 2020), both characterized by the presence of the angiotensin-converting enzyme-2 (ACE2) receptor used by the viral Spike protein to penetrate inside the human cells (Li et al. 2020). In the direct route (“the olfactory route”), once reached the nasal cavity, viruses adhere to the nasal mucosa and through the olfactory sensory neurons move to the olfactory nerve, and, crossing the blood–brain barrier, enter the brain. Furthermore, the abundance of blood vessels and lymphatics in the mucosa favors the brain invasion through the bloodstream (“the hematogenous route”). The nasal mucosa and the eye conjunctiva are also the doors for the trigeminal nerve and hence for the brain (Li et al. 2020). It has been done the hypothesis that the brain invasion through the indirect way starts at the level of the ACE2 receptors present in the lung epithelium from which the virus enters the bloodstream, infects the blood–brain barrier and spreads into the brain (Li et al. 2020). Once entered the CNS, because of the diffuse presence of ACE2 receptors, SARS-CoV-2 can cause disruption of the normal brain homeostasis (Braga et al. 2022), mitochondrial dysfunction, microglia activation, persistent neuroinflammation, and protein misfolding and aggregation leading to cell death (Song et al. 2021). It is interesting to observe that these injuries are shared with some neurodegenerative diseases such as Alzheimer’s disease (AD) and Parkinson’s disease (PD), optic neuritis and multiple sclerosis. Of interest, some authors have advanced the hypothesis that SARS-CoV-2 infection can cause the development or progression of neurodegenerative diseases (Light 2022; Zarifkar et al. 2022). Once again, the link between SARS-CoV-2-induced disorders and neurodegenerative diseases is being questioned. Moreover, viruses belonging to the Coronavirus family have been found in patients affected by such neurodegenerative disorders suggesting that the prolonged coronavirus infection may contribute to the insurgence of these neuropathological diseases. Recently, some Authors have proposed that cross-reactive antibodies generated after SARS-CoV-2 infection may explain the insurgence of the long-COVID. The same authors suggested that vaccination trials should consider the formation of tissue reactive antibodies (Kreye et al. 2020). However, while SARS-CoV-2 has been detected in brain of severely COVID-19 patients, there are no clear evidence about the route followed to reach the CNS structures, although diffuse signs of inflammation were present (McQuaid et al. 2022).

At the molecular level, SARS-CoV-2 and the human cells interact through the direct binding between the viral surface protein Spike (S) and the human ACE2 highly expressed in lungs, heart, kidneys, and adipose tissue (Nuzzo et al. 2021a). Further action of the human proteases is also needed to allow the entrance of the virus. The S protein presents two domains: S1 and S2. The C and N protein terminals are included in the S1 domain. The Receptor Binding Domain (RBD), responsible for the binding to the host cell, is in the C-terminal region (Nuzzo et al. 2021a).

Overall, the neurological involvement associated with COVID-19 is very common and multifaceted and could pose relevant issues for prognosis and treatment. Determinants of cognitive impairment/brain fog in subjects with COVID-19 are still not well characterized. A recent large cross-sectional study, including over seven-hundred individuals aged 18 years or older, found that about 20% of subjects experienced cognitive impairment several months after COVID-19 infection (Becker et al. 2021).

The cognitive impairment pattern was suggestive of a *dysexecutive syndrome*, with impairment of attentive and executive functioning. These data were confirmed and strengthened by a recent positron emission tomography imaging study, which revealed a predominant frontoparietal hypometabolism in patients with subacute COVID-19 infection (Hosp et al. 2021). Another relevant issue is the evaluation of the effect of COVID-19 quarantine in subjects with the prodromal phase of dementia, the so-called Mild Cognitive Impairment (MCI). Indeed, in these subjects, social distancing and isolation due to lockdown led to worsening of cognitive and motor functioning and neuropsychiatric symptoms in over one-third of patients (Baschi et al., 2020). Taken together, all the above data will have relevant implications for cognitive rehabilitation programs and psychological and social interventions.

## Psychiatric Sequelae and Psychological Care in Long-COVID-19 Patients

As mentioned in the previous paragraph, COVID-19 infection has also had a negative effect on mental health. In particular, the most frequent psychiatric manifestations during COVID-19 seem to be sleep disorders, anxiety, and depression (Premraj et al. 2022). Of interest, these disorders increase significantly in prevalence over time.

Researchers conducted in France, Italy, USA, and China indicated that more than 84% of the patients discharged from hospital after SARS-CoV-2 infection showed—in addition to neurological complications—high level of psychological distress, post-traumatic stress disorder, anxiety, depression, and concentration and sleep abnormalities (Nalbandian et al. 2021). Furthermore, a Chinese study indicated that women are more susceptible to experience fatigue, anxiety, and depression up to 6 months after the negative swab (Nalbandian et al. 2021).

Since its first appearance, the diffusion of the new SARS-Cov-2 virus scared and worried people worldwide, especially when it began to be understood that the effects of the disease could have been far worse than initially thought. The hard measures taken worldwide by many Governments to control and limit the pandemic diffusion, such as lockdown, isolation, and maintenance of the social distances have had, therefore, a decline of the general well-being, with the heaviest effects on women and young people (Ai et al. 2021). An investigation conducted between the fall of 2020 and the spring of 2021 individuated in psychological distress, self-harm, subjective well-being, and loneliness were the four descriptors to study the psychological state of the population (Aknin et al. 2022; Azimi Green and Zeller Manley 2022). Although the research is conducted with very rigorous methodology, the same authors invite to consider two fundamental points of discussion, that is the fast evolution of the disease landscape and the greater availability and reliability of data related to the Western countries. The latter point makes difficult to consider the results generalizable to other Nations (Aknin et al. 2022). Concerning psychological distress described by anxiety and depression, the authors found a threefold (30.7%) and double (27.5%) increase of the percentage of people affected by such disorders, respectively, compared with data obtained before pandemic (10.2% and 14.7%). In the United Kingdom, the self-harm propensity was increased from 4.5% to 18%. The well-being feeling showed a clear decrease in several Western countries. The percentage of people complaining of loneliness rose from 2% in the pre-pandemic period to 18% by June 2020 (Aknin et al. 2022). Investigation of 205 consecutive COVID-19 patients up to 6-month post infection indicated increased anxiety, low mood, and depression, especially in hospitalized patients. Overall, one-third of them did not recover their pre-infection health status (Holdsworth et al. 2022). The authors, however, caution against considering these results as general because they were obtained in predominantly male, young people, and members of the British armed forces. Braga et al. found a high incidence of neuropsychological problems among female vs male in a sample of 614 adults (Braga et al. 2022). The prolonged confinement due to the COVID-19 lockdown, has had a deleterious impact particularly on the mental health of young people up to 20 years old, particularly if disadvantaged (Creswell et al. 2021). The young people had to stop their activities, sports, hanging out with friends, school, and university. Furthermore, uneventful or even violent family relationships and the lack of work have further aggravated their psychological distress (Crawford 2021). In United States, an investigation performed in 2020, before the vaccine development and administration, on about 900 participants in the age range 18–30 years, reported that about half of the participants presented high levels of depression (43%) and anxiety (45.4%), while more than one-third reported symptoms configurable with the Post Traumatic Stress Syndrome (PTSD). Interestingly, compared to whites’, Asian Americans described a lower degree of mental problems, while Hispanics were less likely to have anxiety problems (Liu et al. 2020a).

## Physical Activity in Long-COVID-19 Patients

According to a recent report on the prevalence of sarcopenia in intensive care units, some research documented that after the COVID-19 acute phase, 58% of the patients complained of sarcopenia problems and significant reduction in body mass. Sarcopenia can develop in a variable time from 1 to 6 months and beyond. The extent of the muscle loss is influenced by several factors. One of the causes originating sarcopenia in COVID-19 infection is linked to the anomalous development of pro-inflammatory cytokines. Particularly in the older subjects, the latter could influence the changes occurring in the body affecting the skeleton and the muscular tissue during SARS-CoV-2 infection (Piotrowicz et al. 2021). Then, how to alleviate the negative consequences of the pathology on mental and physical health? The answer to this question could be to favor the regular practice of physical exercise. The latter, in addition to having a fundamental function during rehabilitation, has the ability to alleviate psychological suffering, thus improving the quality of life (Nieman 2021). Physical exercises decrease the levels of stress hormones, such as adrenaline and cortisol, and increase the concentration of positive hormones, endorphins, and brain-derived neurotrophic factors making people happy, relaxed, and optimistic (Harber and Sutton 1984).

The fundamental role of physical exercise for long-COVID patient rehabilitation has been reported. The US Guideline of physical activity for adults recommend a moderate-intensity exercise weekly (McGregor et al. 2021). Also under chronic medical conditions, physical activity is strongly suggested (Piercy et al. 2018). 150-to-300 min a week represents an optimum time for exercise (Piercy et al. 2018). Emerged data suggest that daily exercise may contribute also to reduce acute respiratory distress syndrome in healthy people, and in COVID-19 and long-COVID patients (Nyenhuis et al. 2020). The COVID-19 pandemic has modified the way people approach physical activity. In many countries, gyms have been closed as a measure to prevent the spread of infections. Consequently, home fitness and outdoor activities have increased in popularity because of more safety and these choices look to be the new normality in the next future. Yoga, pilates, and aerobic exercises do not require particular equipment and space and can be easily practiced at home. Aerobic exercise reduces the risk of many health conditions, ranging from heart disease to brain disorders. While all forms of physical activity provide some benefits, aerobic exercise is particularly effective because it makes the brain, heart, and lungs work harder than usual (Guiney and Machado 2013). The nutritional regimen can also help in improving emotional health. Several researches indicated that the assumption of plant-based foods, particularly those ones containing carotenoids, is associated with a well-being feeling and optimism.

## Long-COVID-19, Dysmetabolism, and Alimentary Regimen

Nutrition is closely linked to long-COVID because nutrition disorders are a risk factor for the onset of the disease and adequate nutrition is strictly recommended during its course. Long-COVID-19 prevails on specific groups of population, particularly overweight, obese, and diabetic subjects. Obesity influences the inter-systemic homeostasis involving the endocrine, neurological, and immune–metabolic systems. This might explain the vulnerability of these subjects to infectious risks. A recent study conducted by Aminian et al. on 2,839 COVID-19 patients who survived the acute phase without hospitalization showed that 8 months after acute disease, the risk of hospitalization was, respectively, 28% and 30% higher in patients with moderate and severe obesity (BMI ≥ 35) compared to patients with a normal body mass index (BMI < 30) (Aminian et al. 2021). However, another research has shown that in some cases of non-critically ill hospitalized patients affected by COVID-19, a high value of BMI can be a protective factor and that it is the presence of other pathologies to determine, instead, the negative contribution of the high body weight (Caccialanza et al. 2021a).

Metabolic dysfunction and, particularly, type 2 diabetes mellitus (T2DM) are often associated with high mortality, great severity, and worse progression of COVID-19 (Apicella et al. 2020). This association depends on different reasons: the overexpression of ACE2 receptor in individuals with diabetes, comorbidities such as hypertension and cardiovascular disease, obesity, and pro-inflammatory state (Apicella et al. 2020). Age, sex, and ethnicity play also a role in the disease outcome (Apicella et al. 2020). In a recent study pain or sleep disturbances have been identified in diabetic patients 4 weeks after the acute phase of the COVID-19 disease (Akter et al. 2020). The COVID-19 pandemic has also resulted in inadequate diabetes management, an increase in the number of new diabetes and corticosteroid-induced diabetes cases.

Although it is well recognized that nutrition has a significant role in individual health, only few data have described the role of adequate alimentary regimen during long-COVID disease. A recent investigation conducted among 164 COVID-19 patients compared with 183 controls showed the persistence of mild gastroenterological symptoms, in particular in patients reporting diarrhea in the acute phase of the infection, 5 months after the acute infection (Noviello et al. 2022). Infected patients are also at increased risk of chronic fatigue and somatoform disorders, supporting the hypothesis that functional gastrointestinal and somatoform disorders may have a common biological origin (Noviello et al. 2022). Furthermore, several studies have indicated that the gastrointestinal tract is highly involved in the long-term effects of SARS-CoV-2 infection with enhanced expression of the ACE2 receptors and gut microbiota modifications with increased presence of opportunistic pathogens and loss of beneficial microbiota, especially of those having an immunomodulatory activity (Liu et al. 2022). More and more evidences indicate anomalous immunological signs affecting the gastrointestinal apparatus with inflammation, possibly due to auto-immunity. Furthermore, viral persistence, microbial dysbiosis, and altered neuro-immune interactions in the gut may contribute to the pathogenesis of gastrointestinal long-COVID (Meringer and Mehandru 2022).

It has been observed that the long-COVID shares some symptoms with the already documented pathology known as myalgic encephalomyelitis/chronic fatigue symptoms (ME/CFS). The latter is an unexplained post-viral disorder associated with infections such as Epstein–Barr or glandular fever, affecting 0.2–2% of individuals worldwide (Wood et al. 2021). A large number of COVID-19 patients around the world suggests that long-COVID could affect a large number of individuals. For this reason, it is important to develop protocols to reduce the discomfort of sick people. The existence of some common aspects between ME/CFS and long-COVID, such as oxidative stress and mitochondrial dysfunction, suggests that food or nutritional supplements containing antioxidants can be a viable route also in the case of the latter pathology (Wood et al. 2021). In fact, the cytokine storm, occurring in COVID-19, causes oxidative and inflammatory states and antioxidants, obtained by spices, herbs, roots, fruits, and vegetables can reduce the insurgence of severe disease and give strength to the immune system. There is growing evidence, from animal studies and human clinical trials, that diets rich in anthocyanins protect against inflammation and increase gut permeability as well as improve colon health through their ability to alter bacterial metabolism and microbial milieu within the intestines (Li et al. 2019).

Besides the well-known polyphenols derived from fruit and vegetables, biologically active and useful substances have been identified in eggs: several egg white proteins, including ovalbumin, ovotransferrin, ovomucin, lysozyme, and avidin, as well as the peptides derived from the proteins, have been recognized for their functional importance as antimicrobial, antioxidant, and anti-inflammatory agents and for their ACE-inhibitory activity (Yu et al. 2014).

The role of vitamins and minerals for a correct immune system response has been fully investigated. Many nutrients, such as vitamin A, B, C, and D, and minerals like zinc, and selenium play a key role in maintaining a healthy immune system. Vitamin A is fundamental for the performances of T and B cells and for antibody production and its deficiency has been associated in vivo with reduced number and functionality of natural killer cells, activity of phagocytic cells and macrophages (Chang and Hou 2015). Vitamins of group B assist in the inflammatory processes, lymphocyte proliferations and their deficiency can significantly affect the immune system response (Arruda de Souza Monnerat et al. 2020). Vitamin C has prevalently an antioxidant and immunomodulatory effects and protects lymphocytes from oxidative stress (Arruda de Souza Monnerat et al. 2020). Vitamin D supplementation has been suggested to have positive effects on immune-stimulation against infectious viruses. People affected by COVID-19 present an insufficient vitamin D level and its reduced level had also been identified in the coronavirus infection of bovines. Furthermore, Hayashi and collaborators showed that vitamin D supplementation in mouse model mitigated the clinical manifestations of influenza virus infection by suppressing virus replication and inflammation (Hayashi et al. 2020). However, despite the low concentration levels of vitamin D in COVID-19-affected patients, there is still no clear evidence of a correlation between vitamin D administration and positive disease outcomes (Caccialanza et al. 2021b).

Zinc is an essential micronutrient that regulates different immunity functions. It plays a crucial role in viral infections. Increased concentration levels of intracellular zinc by zinc ionophores like pyrithione can efficiently weaken the replication of a variety of RNA viruses (te Velthuis et al. 2010). The combination of zinc with pyrithione could inhibit SARS coronavirus replication (te Velthuis et al. 2010). Another important micronutrient involved in the immune response is selenium. This element is essential for regulation of mammalian redox biology. Selenium assists a group of enzymes that, in concert with vitamin E, work to prevent the formation of free radicals and oxidative cell and tissue damages. Selenium deficiency induces impairment of the host immune system and rapid mutation of benign RNA viruses to the virulent forms.

Flavonoids are a group of natural substances that have different subgroups including chalcones, flavonols, flavones, and isoflavones. These molecules have many activities besides antioxidant effects and antiviral capacity. Recent research has suggested that the anti-coronavirus activity of some flavonoids (herbacetin, rhoifolin, and pectolinarine) is due to the inhibition of the 3C-like protease (Jo et al. 2020). Furthermore, herbacetin, quercetin, and helichrysetin were found to be able to block the MERS-CoV/3CL pro enzymatic activity (Jo et al. 2020).

In conclusion, the use of a plant-based diet and of freshly cooked and lightly processed foods, avoiding bioactive compound degradation, could help in reducing the symptoms related to long-COVID. Furthermore, data collected before the pandemic era indicated that a vegetable alimentary regimen usually reduced symptoms found in long-COVID patients such as sleep disorder, anxiety headaches, and depression.

## Recommendations

Many are the protocols drafted worldwide to reduce the virus spreading and the risk connected with COVID-19 and long-COVID-19 diseases (Yelin et al. 2022; Berlit et al. 2020; Azimi Green and Zeller Manley 2022). However, due to the very new pathology, there is not enough scientific evidence to make specific recommendations. Guidelines only provide evidence-based recommendations for assessing and managing people with long-COVID-19, providing a definition of this condition. The rehabilitation process for COVID-19 and long-COVID-19 requires a multidisciplinary approach strictly connected to the patient's needs. In particular, treatment of long-COVID-19 patients involves two aspects: respiratory function and physical re-education (Carda et al. 2020). Physical exercise and aerobic training, together with neuromotor and peripheral muscle rehabilitation activities, proved to be fundamental in the pulmonary therapy (Liu et al. 2020b). In contrast, there are no specific drug treatments for the neurological effects of the long-COVID-19 (Yelin et al. 2022). Some articles propose therapeutic interventions but they did not provide any clinical evidence. The use of the flavonoid luteolin, which inhibits the pro-inflammatory cascade of mast cells and microglia, has been suggested as a potential treatment (Theoharides et al. 2021). From simulation and molecular docking studies, cannabis has been shown to have the potential to bind and downregulate central nervous system proteins linked to symptoms of long-COVID-19 (Sarkar et al. 2021). Methylene blue, due to its mitochondrial protective effects, has been suggested as a possible therapy against neurological deterioration due to SARS-CoV-2 (Magoon et al. 2021). The hormone melatonin has been identified as an activator of erythroid-derived nuclear factor 2-like 2 (NRF2). NRF2 can increase the expression of glutathione, which extinguishes the free radicals that, in turn, cause oxidative stress (Jarrott et al. 2022). Therefore, melatonin is a possible drug to be considered in long-COVID-19 patients suffering from fatigue, insomnia, depression, and “brain fog” (Jarrott et al. 2022) but appropriate clinical trials designed to evaluate melatonin are needed (Jarrott et al. 2022). Furthermore, natural substances such as grapefruit seed extracts and their main components, belonging to the limonoids class, have been tested for their antioxidant activity and for counteracting the COVID-19 (Magurano et al. 2021). Regarding the emotional and psychiatric consequences of the long-COVID-19, the use of clomipramine, a tricyclic antidepressant with an anti-inflammatory action able to penetrate the brain has been suggested (Nobile et al. 2021). At the moment, these data are only theoretical or supported by in vitro experiments. A further step will be taken when the molecular basis underlying the long-Covid-19 will better clarified.

## Conclusions

**Fig. 2:**
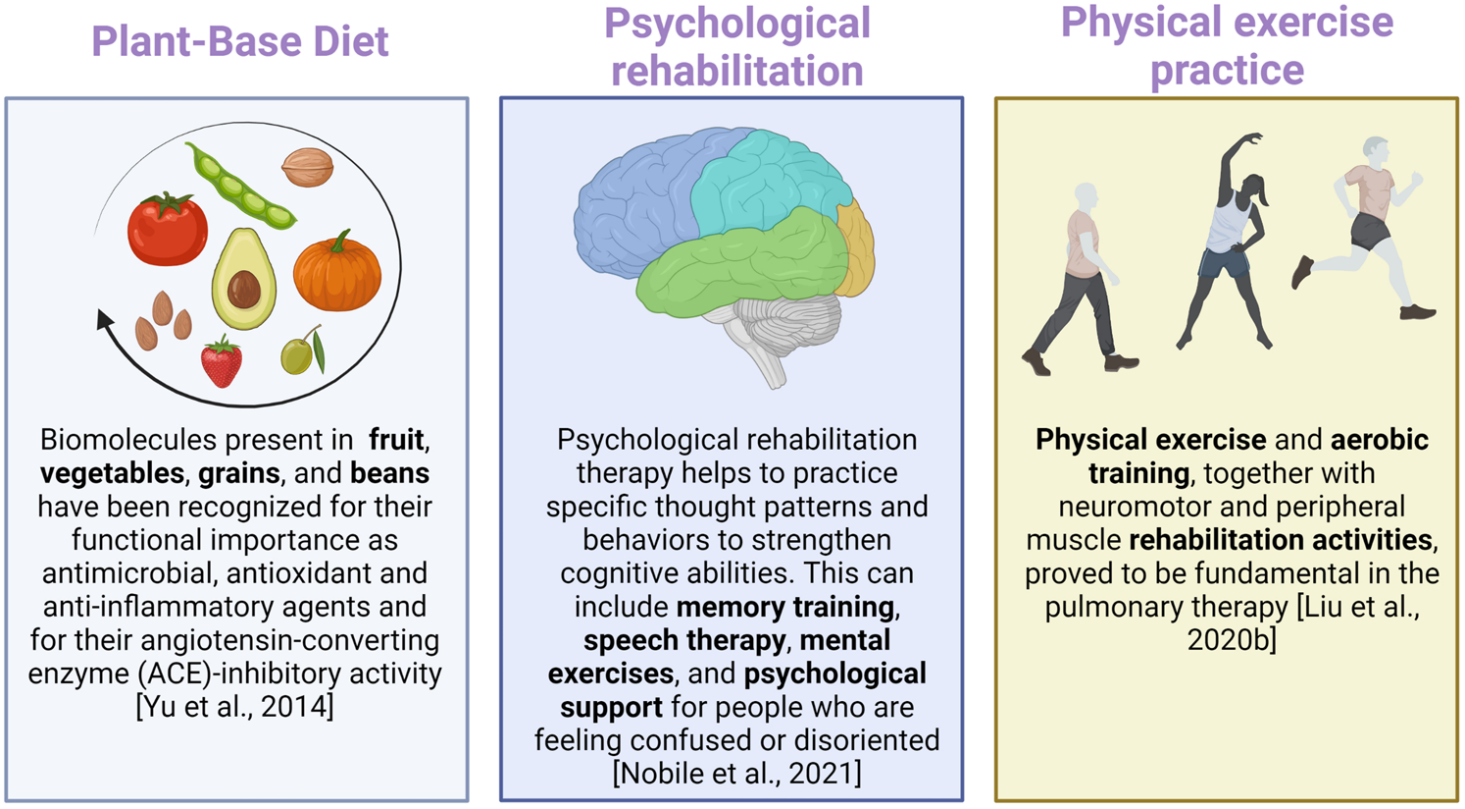
Intervention plan for long-COVID home management. Image created with BioRender.com

Resolving the cognitive, psychological, and physical consequences of COVID-19 and long-COVID diseases requires a multidisciplinary approach for a period that is very difficult to determine now. This approach must be tailored considering the individual characteristics of a patient. For these reasons, an appropriate diet, cognitive and psychological support, and correct physical activity may be useful for a rapid improvement of the syndrome (Fig. 2). Recently ongoing multidimensional trials, such as the Rehabilitation Exercise and psycholoGical support After covid-19 InfectioN (REGAIN), will be helpful to evaluate and manage the impact of long-COVID in the population.
